# Crystal structure of 2′-hy­droxy­aceto­phenone 4-methyl­thio­semicarbazide

**DOI:** 10.1107/S2056989015004958

**Published:** 2015-03-18

**Authors:** Junita Jamsari, Nur Fatihah Abas, Thahira Begum S. A. Ravoof, Edward R. T. Tiekink

**Affiliations:** a Department of Chemistry, Universiti Putra Malaysia, 43400 Serdang, Malaysia; b Department of Chemistry, University of Malaya, 50603 Kuala Lumpur, Malaysia

**Keywords:** crystal structure, thio­semicarbazide, hydrogen bonding, O—H⋯π inter­actions

## Abstract

In the organic mol­ecule of the title hydrate, C_11_H_15_N_3_OS·H_2_O, {systematic name: 3-ethyl-1-{(*E*)-[1-(2-hy­droxy­phen­yl)ethyl­idene]amino}­thio­urea monohydrate}, a dihedral angle of 5.39 (2)° is formed between the hy­droxy­benzene ring and the non-H atoms comprising the side chain (r.m.s. deviation = 0.0625 Å), with the major deviation from planarity noted for the terminal ethyl group [the C—N—C—C torsion angle = −172.17 (13)°]. The N—H H atoms are *syn* and an intra­molecular hy­droxy–imine O—H⋯N hydrogen bond is noted. In the crystal, the N-bonded H atoms form hydrogen bonds to symmetry-related water mol­ecules, and the latter form donor inter­actions with the hy­droxy O atom and with a hy­droxy­benzene ring, forming a O—H⋯π inter­action. The hydrogen bonding leads to supra­molecular tubes aligned along the *b* axis. The tubes are connected into layers *via* C—H⋯O inter­actions, and these stack along the *c* axis with no directional inter­actions between them.

## Related literature   

For background to thio­semicarbazones and their coordination chemistry, see: Mazlan *et al.* (2014 ▸). The conformational flexibility in these mol­ecules is reflected in the structure of the 4-methyl derivative where one mol­ecule comprising the asymmetric unit is approximately planar and the other exhibits a clear twist between the hy­droxy­benzene and side chain, and in the structure of the 6-meth­oxy derivative where these residues are almost normal to each other, see: Anderson *et al.* (2012 ▸, 2014 ▸). For synthesis and methodology, see: Omar *et al.* (2014 ▸).
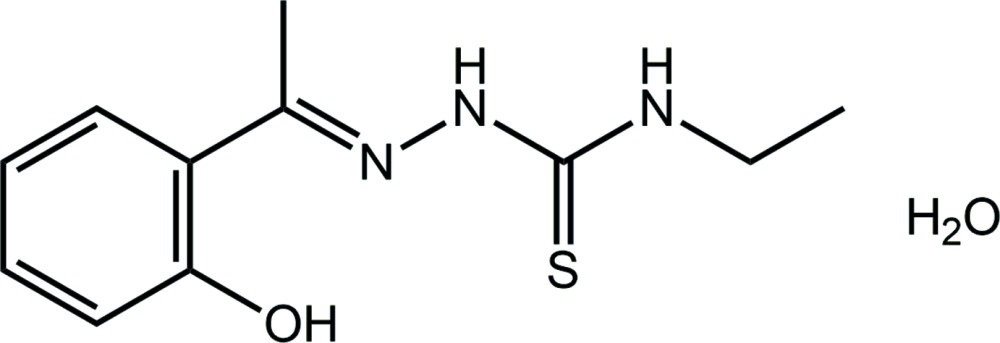


## Experimental   

### Crystal data  

C_11_H_15_N_3_OS·H_2_O
*M* *_r_* = 255.33
Triclinic, 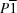

*a* = 6.7947 (5) Å
*b* = 8.5169 (8) Å
*c* = 11.1199 (9) Å
α = 84.948 (7)°
β = 81.825 (6)°
γ = 84.084 (7)°
*V* = 631.81 (9) Å^3^
*Z* = 2
Cu *K*α radiation
μ = 2.25 mm^−1^
*T* = 100 K
0.30 × 0.20 × 0.10 mm

### Data collection  

Oxford Diffraction Xcaliber Eos Gemini diffractometer
Absorption correction: multi-scan (*CrysAlis PRO*; Agilent, 2011 ▸) *T*_min_ = 0.923, *T*_max_ = 1.000
8119 measured reflections
2408 independent reflections
2187 reflections with *I* > 2σ(*I*)
*R*_int_ = 0.026

### Refinement  

*R*[*F* ^2^ > 2σ(*F* ^2^)] = 0.035
*wR*(*F* ^2^) = 0.095
*S* = 1.03
2408 reflections
171 parameters
6 restraints
H atoms treated by a mixture of independent and constrained refinement
Δρ_max_ = 0.33 e Å^−3^
Δρ_min_ = −0.25 e Å^−3^

### 

Data collection: *CrysAlis PRO* (Agilent, 2011 ▸); cell refinement: *CrysAlis PRO*; data reduction: *CrysAlis PRO*; program(s) used to solve structure: *SHELXS97* (Sheldrick, 2015 ▸); program(s) used to refine structure: *SHELXL2014* (Sheldrick, 2015 ▸); molecular graphics: *ORTEP-3 for Windows* (Farrugia, 2012 ▸) and *DIAMOND* (Brandenburg, 2006 ▸); software used to prepare material for publication: *publCIF* (Westrip, 2010 ▸).

## Supplementary Material

Crystal structure: contains datablock(s) 1, I. DOI: 10.1107/S2056989015004958/hb7380sup1.cif

Structure factors: contains datablock(s) I. DOI: 10.1107/S2056989015004958/hb7380Isup2.hkl

Supporting information file. DOI: 10.1107/S2056989015004958/hb7380Isup3.cml

**Fig. 1.**
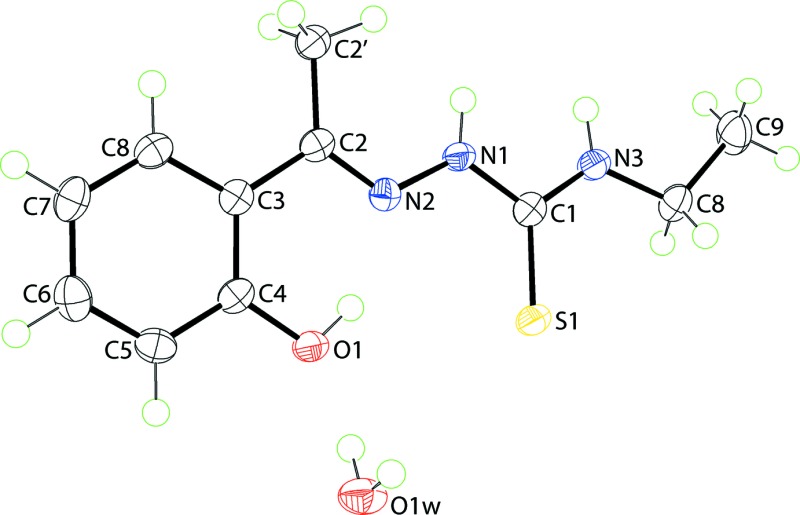
The molecular structure of the title compound showing the atom-labelling scheme and displacement ellipsoids at the 70% probability level.

**Fig. 2.**
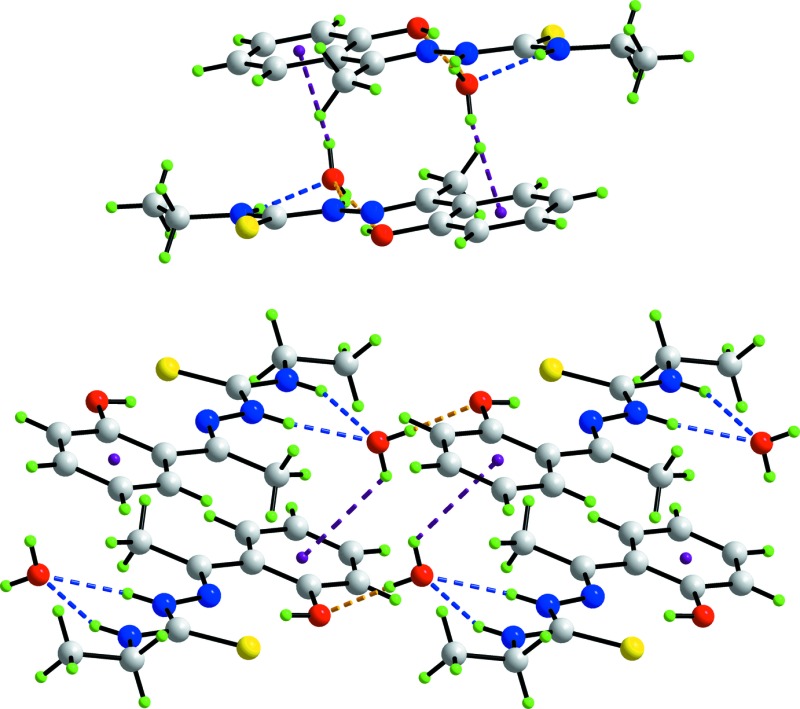
Two views of the supramolecular tube along the b axis sustained by O—H···O, N—H···O and O—H···π hydrogen bonding, shown as orange, blue and purple dashed lines, respectively.

**Fig. 3.**
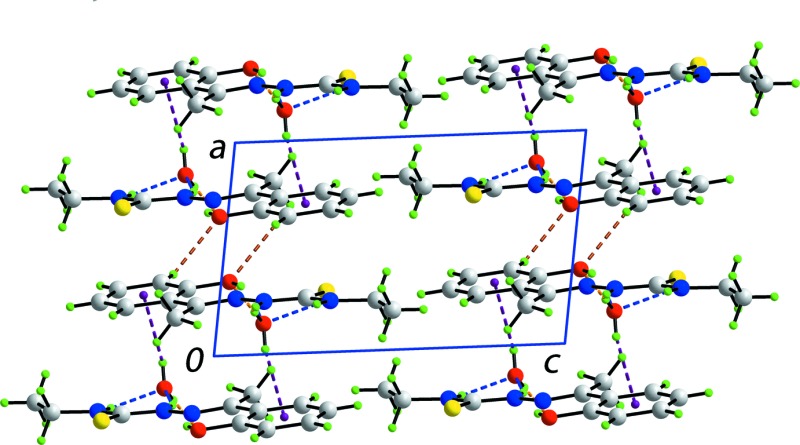
A view of the unit-cell contents in projection down the b axis. The O—H···O, N—H···O, O—H···π and C—H···O interactions are shown as orange, blue, purple and brown dashed lines, respectively.

. DOI: 10.1107/S2056989015004958/hb7380fig1.tif
The mol­ecular structure of the title compound showing the atom-labelling scheme and displacement ellipsoids at the 70% probability level.

b . DOI: 10.1107/S2056989015004958/hb7380fig2.tif
Two views of the supra­molecular tube along the *b* axis sustained by O—H⋯O, N—H⋯O and O—H⋯π hydrogen bonding, shown as orange, blue and purple dashed lines, respectively.

b . DOI: 10.1107/S2056989015004958/hb7380fig3.tif
A view of the unit-cell contents in projection down the *b* axis. The O—H⋯O, N—H⋯O, O—H⋯π and C—H⋯O inter­actions are shown as orange, blue, purple and brown dashed lines, respectively.

CCDC reference: 1053189

Additional supporting information:  crystallographic information; 3D view; checkCIF report

## Figures and Tables

**Table 1:** Hydrogen-bond geometry (, ) *Cg*1 is the centroid of the C3C8 ring.

*D*H*A*	*D*H	H*A*	*D* *A*	*D*H*A*
O1H1*O*N2	0.84(1)	1.76(1)	2.5292(16)	150(2)
N1H1*N*O1*W* ^i^	0.86(2)	2.09(2)	2.8918(17)	156(2)
N3H3*N*O1*W* ^i^	0.87(2)	2.16(2)	2.9625(18)	154(2)
O1*W*H1*W*O1	0.84(2)	1.96(2)	2.7894(16)	174(2)
O1*W*H2*W* *Cg*1^ii^	0.83(1)	2.86(1)	3.4165(13)	127(1)
C5H5O1^iii^	0.95	2.56	3.3702(18)	143

Symmetry codes: (i) 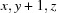; (ii) 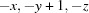; (iii) 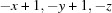.

## supplementary crystallographic information

### S1. Experimental

4-Methyl-3-thiosemicarbazide (0.02 mol) was dissolved in hot ethanol (95%; 40 ml). A solution of 0.02 mol of 2'-hydroxyacetophenone was added drop-wise into the first solution. The mixture was heated and stirred to reduce the volume to half of its initial volume. Then, it was allowed to stand at room temperature until a white crystalline precipitate formed. The precipitate was then collected and recrystallized from ethanol and dried over silica gel. Colourless crystals were obtained from the ethanolic solution. Yield: 92%. *M*.pt: 116 °C. Anal. Found (Calc): C: 56.1 (55.7); H: 5.9 (6.4); N: 18.5 (17.7).

### S2. Refinement

Carbon-bound H-atoms were placed in calculated positions (C—H = 0.95 to 0.99 Å) and were included in the refinement in the riding model approximation with *U_iso_*(H) = 1.2–1.5*U_eq_*(C). The O—H H atoms were refined with O—H = 0.84±0.01 Å, and with *U_iso_*(H) = 1.5*U_eq_*(O). The N—H H atoms were refined similarly with N—H = 0.88±0.01 Å, and with *U_iso_*(H) = 1.2*U_eq_*(N).

### Crystal data

**Table:**

C_11_H_15_N_3_OS·H_2_O	*Z* = 2
*M**_r_* = 255.33	*F*(000) = 272
Triclinic, *P*1	*D*_x_ = 1.342 Mg m^−^^3^
*a* = 6.7947 (5) Å	Cu *K*α radiation, λ = 1.5418 Å
*b* = 8.5169 (8) Å	Cell parameters from 4230 reflections
*c* = 11.1199 (9) Å	θ = 4.0–71.4°
α = 84.948 (7)°	µ = 2.25 mm^−^^1^
β = 81.825 (6)°	*T* = 100 K
γ = 84.084 (7)°	Prism, pale-yellow
*V* = 631.81 (9) Å^3^	0.30 × 0.20 × 0.10 mm

### Data collection

**Table:**

Oxford Diffraction Xcaliber Eos Gemini diffractometer	2408 independent reflections
Radiation source: fine-focus sealed tube	2187 reflections with *I* > 2σ(*I*)
Graphite monochromator	*R*_int_ = 0.026
Detector resolution: 16.1952 pixels mm^-1^	θ_max_ = 71.5°, θ_min_ = 4.0°
ω scans	*h* = −8→8
Absorption correction: multi-scan (*CrysAlis PRO*; Agilent, 2011)	*k* = −10→9
*T*_min_ = 0.923, *T*_max_ = 1.000	*l* = −13→13
8119 measured reflections	

### Refinement

**Table:**

Refinement on *F*^2^	6 restraints
Least-squares matrix: full	Hydrogen site location: mixed
*R*[*F*^2^ > 2σ(*F*^2^)] = 0.035	H atoms treated by a mixture of independent and constrained refinement
*wR*(*F*^2^) = 0.095	*w* = 1/[σ^2^(*F*_o_^2^) + (0.0593*P*)^2^ + 0.1686*P*] where *P* = (*F*_o_^2^ + 2*F*_c_^2^)/3
*S* = 1.03	(Δ/σ)_max_ < 0.001
2408 reflections	Δρ_max_ = 0.33 e Å^−^^3^
171 parameters	Δρ_min_ = −0.25 e Å^−^^3^

### Special details

**Table:**

Geometry. All e.s.d.'s (except the e.s.d. in the dihedral angle between two l.s. planes) are estimated using the full covariance matrix. The cell e.s.d.'s are taken into account individually in the estimation of e.s.d.'s in distances, angles and torsion angles; correlations between e.s.d.'s in cell parameters are only used when they are defined by crystal symmetry. An approximate (isotropic) treatment of cell e.s.d.'s is used for estimating e.s.d.'s involving l.s. planes.

### Fractional atomic coordinates and isotropic or equivalent isotropic displacement parameters (Å^2^)

**Table:**

	*x*	*y*	*z*	*U*_iso_*/*U*_eq_	
S1	0.28553 (5)	0.84923 (4)	0.30332 (3)	0.01932 (14)	
O1	0.34603 (16)	0.67711 (12)	0.02373 (9)	0.0193 (2)	
H1O	0.325 (3)	0.7637 (15)	0.0568 (16)	0.029*	
N1	0.24861 (18)	1.08372 (15)	0.12735 (11)	0.0163 (3)	
H1N	0.224 (3)	1.1825 (17)	0.1053 (16)	0.020*	
N2	0.26593 (17)	0.97046 (14)	0.04679 (11)	0.0150 (3)	
N3	0.24119 (18)	1.16243 (16)	0.31656 (11)	0.0174 (3)	
H3N	0.227 (3)	1.2558 (18)	0.2795 (15)	0.021*	
C1	0.2583 (2)	1.03944 (18)	0.24810 (13)	0.0160 (3)	
C2	0.2391 (2)	1.00570 (18)	−0.06558 (13)	0.0153 (3)	
C2'	0.1872 (2)	1.16980 (18)	−0.12094 (13)	0.0192 (3)	
H2'1	0.1497	1.2423	−0.0561	0.029*	
H2'2	0.0750	1.1686	−0.1672	0.029*	
H2'3	0.3030	1.2053	−0.1755	0.029*	
C3	0.2674 (2)	0.86995 (18)	−0.14260 (13)	0.0154 (3)	
C4	0.3216 (2)	0.71390 (18)	−0.09556 (13)	0.0164 (3)	
C5	0.3532 (2)	0.58929 (18)	−0.17128 (14)	0.0193 (3)	
H5	0.3914	0.4854	−0.1391	0.023*	
C6	0.3291 (2)	0.61601 (19)	−0.29349 (14)	0.0214 (3)	
H6	0.3503	0.5303	−0.3445	0.026*	
C7	0.2739 (2)	0.76788 (19)	−0.34155 (13)	0.0206 (3)	
H7	0.2562	0.7863	−0.4250	0.025*	
C8	0.2451 (2)	0.89188 (19)	−0.26665 (13)	0.0179 (3)	
H8	0.2089	0.9955	−0.3004	0.021*	
C9	0.2364 (2)	1.14844 (19)	0.44845 (13)	0.0202 (3)	
H9A	0.3678	1.1022	0.4697	0.024*	
H9B	0.1344	1.0773	0.4859	0.024*	
C10	0.1872 (3)	1.3101 (2)	0.49712 (14)	0.0271 (4)	
H10A	0.2892	1.3797	0.4605	0.041*	
H10B	0.1840	1.3003	0.5858	0.041*	
H10C	0.0563	1.3549	0.4766	0.041*	
O1W	0.14391 (17)	0.42112 (13)	0.13237 (11)	0.0243 (3)	
H1W	0.200 (3)	0.5018 (17)	0.1042 (17)	0.036*	
H2W	0.0232 (16)	0.452 (2)	0.1447 (19)	0.036*	

### Atomic displacement parameters (Å^2^)

**Table:**

	*U*^11^	*U*^22^	*U*^33^	*U*^12^	*U*^13^	*U*^23^
S1	0.0267 (2)	0.0159 (2)	0.01519 (19)	−0.00163 (15)	−0.00399 (14)	0.00149 (14)
O1	0.0279 (6)	0.0139 (5)	0.0164 (5)	−0.0010 (4)	−0.0056 (4)	0.0005 (4)
N1	0.0221 (6)	0.0121 (6)	0.0147 (6)	−0.0017 (5)	−0.0036 (5)	0.0003 (5)
N2	0.0142 (6)	0.0156 (6)	0.0151 (6)	−0.0024 (5)	−0.0014 (4)	−0.0012 (5)
N3	0.0215 (6)	0.0155 (7)	0.0151 (6)	−0.0020 (5)	−0.0033 (5)	0.0005 (5)
C1	0.0126 (6)	0.0191 (8)	0.0166 (7)	−0.0030 (6)	−0.0022 (5)	−0.0002 (6)
C2	0.0126 (6)	0.0168 (8)	0.0165 (7)	−0.0037 (5)	−0.0014 (5)	0.0014 (6)
C2'	0.0239 (7)	0.0174 (8)	0.0164 (7)	−0.0017 (6)	−0.0036 (6)	−0.0003 (6)
C3	0.0120 (6)	0.0179 (8)	0.0164 (7)	−0.0034 (5)	−0.0016 (5)	−0.0007 (6)
C4	0.0146 (6)	0.0187 (8)	0.0162 (7)	−0.0045 (6)	−0.0028 (5)	0.0017 (6)
C5	0.0191 (7)	0.0152 (8)	0.0239 (7)	−0.0037 (6)	−0.0032 (6)	−0.0006 (6)
C6	0.0202 (7)	0.0235 (9)	0.0218 (7)	−0.0070 (6)	−0.0013 (6)	−0.0066 (6)
C7	0.0198 (7)	0.0276 (9)	0.0156 (7)	−0.0063 (6)	−0.0036 (5)	−0.0014 (6)
C8	0.0162 (7)	0.0194 (8)	0.0179 (7)	−0.0027 (6)	−0.0027 (5)	0.0021 (6)
C9	0.0232 (7)	0.0228 (8)	0.0151 (7)	−0.0049 (6)	−0.0028 (5)	−0.0006 (6)
C10	0.0361 (9)	0.0257 (9)	0.0204 (8)	−0.0072 (7)	−0.0004 (6)	−0.0072 (6)
O1W	0.0227 (6)	0.0173 (6)	0.0314 (6)	−0.0025 (5)	−0.0014 (5)	0.0029 (5)

### Geometric parameters (Å, º)

**Table:**

S1—C1	1.6825 (16)	C4—C5	1.393 (2)
O1—C4	1.3653 (17)	C5—C6	1.388 (2)
O1—H1O	0.843 (9)	C5—H5	0.9500
N1—N2	1.3586 (17)	C6—C7	1.391 (2)
N1—C1	1.3713 (18)	C6—H6	0.9500
N1—H1N	0.862 (14)	C7—C8	1.383 (2)
N2—C2	1.2931 (18)	C7—H7	0.9500
N3—C1	1.3344 (19)	C8—H8	0.9500
N3—C9	1.4569 (18)	C9—C10	1.511 (2)
N3—H3N	0.865 (14)	C9—H9A	0.9900
C2—C3	1.480 (2)	C9—H9B	0.9900
C2—C2'	1.505 (2)	C10—H10A	0.9800
C2'—H2'1	0.9800	C10—H10B	0.9800
C2'—H2'2	0.9800	C10—H10C	0.9800
C2'—H2'3	0.9800	O1W—H1W	0.834 (9)
C3—C8	1.403 (2)	O1W—H2W	0.831 (9)
C3—C4	1.417 (2)		
			
C4—O1—H1O	105.5 (13)	C6—C5—C4	120.42 (14)
N2—N1—C1	119.33 (12)	C6—C5—H5	119.8
N2—N1—H1N	121.6 (12)	C4—C5—H5	119.8
C1—N1—H1N	119.0 (12)	C5—C6—C7	120.19 (14)
C2—N2—N1	121.39 (13)	C5—C6—H6	119.9
C1—N3—C9	124.21 (13)	C7—C6—H6	119.9
C1—N3—H3N	116.9 (12)	C8—C7—C6	119.35 (13)
C9—N3—H3N	118.8 (12)	C8—C7—H7	120.3
N3—C1—N1	112.99 (13)	C6—C7—H7	120.3
N3—C1—S1	123.95 (11)	C7—C8—C3	122.27 (14)
N1—C1—S1	123.06 (11)	C7—C8—H8	118.9
N2—C2—C3	115.00 (13)	C3—C8—H8	118.9
N2—C2—C2'	125.29 (13)	N3—C9—C10	109.63 (13)
C3—C2—C2'	119.70 (12)	N3—C9—H9A	109.7
C2—C2'—H2'1	109.5	C10—C9—H9A	109.7
C2—C2'—H2'2	109.5	N3—C9—H9B	109.7
H2'1—C2'—H2'2	109.5	C10—C9—H9B	109.7
C2—C2'—H2'3	109.5	H9A—C9—H9B	108.2
H2'1—C2'—H2'3	109.5	C9—C10—H10A	109.5
H2'2—C2'—H2'3	109.5	C9—C10—H10B	109.5
C8—C3—C4	117.28 (13)	H10A—C10—H10B	109.5
C8—C3—C2	120.87 (13)	C9—C10—H10C	109.5
C4—C3—C2	121.84 (13)	H10A—C10—H10C	109.5
O1—C4—C5	116.68 (13)	H10B—C10—H10C	109.5
O1—C4—C3	122.84 (13)	H1W—O1W—H2W	105 (2)
C5—C4—C3	120.48 (13)		
			
C1—N1—N2—C2	173.45 (12)	C2—C3—C4—O1	1.8 (2)
C9—N3—C1—N1	176.76 (12)	C8—C3—C4—C5	0.8 (2)
C9—N3—C1—S1	−2.4 (2)	C2—C3—C4—C5	−177.99 (12)
N2—N1—C1—N3	179.36 (12)	O1—C4—C5—C6	179.21 (12)
N2—N1—C1—S1	−1.51 (19)	C3—C4—C5—C6	−1.0 (2)
N1—N2—C2—C3	178.75 (11)	C4—C5—C6—C7	0.3 (2)
N1—N2—C2—C2'	0.0 (2)	C5—C6—C7—C8	0.6 (2)
N2—C2—C3—C8	−179.27 (12)	C6—C7—C8—C3	−0.7 (2)
C2'—C2—C3—C8	−0.4 (2)	C4—C3—C8—C7	0.1 (2)
N2—C2—C3—C4	−0.5 (2)	C2—C3—C8—C7	178.85 (13)
C2'—C2—C3—C4	178.29 (12)	C1—N3—C9—C10	−172.17 (13)
C8—C3—C4—O1	−179.39 (12)		

### Hydrogen-bond geometry (Å, º)

Cg1 is the centroid of the C3–C8 ring.

**Table:**

*D*—H···*A*	*D*—H	H···*A*	*D*···*A*	*D*—H···*A*
O1—H1*O*···N2	0.84 (1)	1.76 (1)	2.5292 (16)	150 (2)
N1—H1*N*···O1*W*^i^	0.86 (2)	2.09 (2)	2.8918 (17)	156 (2)
N3—H3*N*···O1*W*^i^	0.87 (2)	2.16 (2)	2.9625 (18)	154 (2)
O1*W*—H1*W*···O1	0.84 (2)	1.96 (2)	2.7894 (16)	174 (2)
O1*W*—H2*W*···*Cg*1^ii^	0.83 (1)	2.86 (1)	3.4165 (13)	127 (1)
C5—H5···O1^iii^	0.95	2.56	3.3702 (18)	143

Symmetry codes: (i) *x*, *y*+1, *z*; (ii) −*x*, −*y*+1, −*z*; (iii) −*x*+1, −*y*+1, −*z*.
