# Supplementary figures and images for: Crystal structure of 2′-hy­droxy­aceto­phenone 4-methyl­thio­semicarbazide
Source: Acta Crystallogr E Crystallogr Commun. 2015 Mar 18;71(Pt 4):o244–5. doi: 10.1107/S2056989015004958 (PMC4438807; doi:10.1107/S2056989015004958)

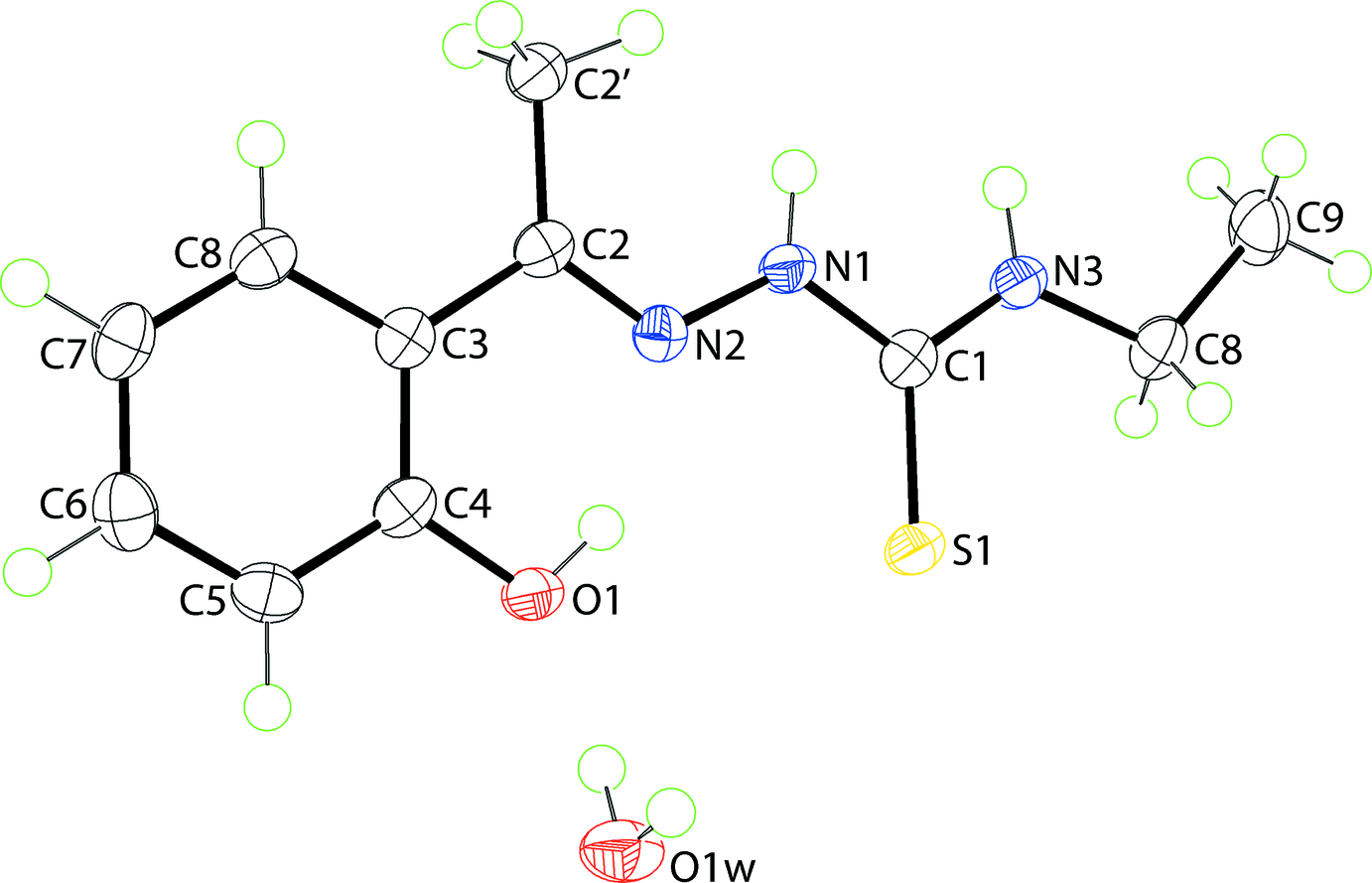

Supplement: Supplementary file 4 [file e-71-0o244-fig1.tif]

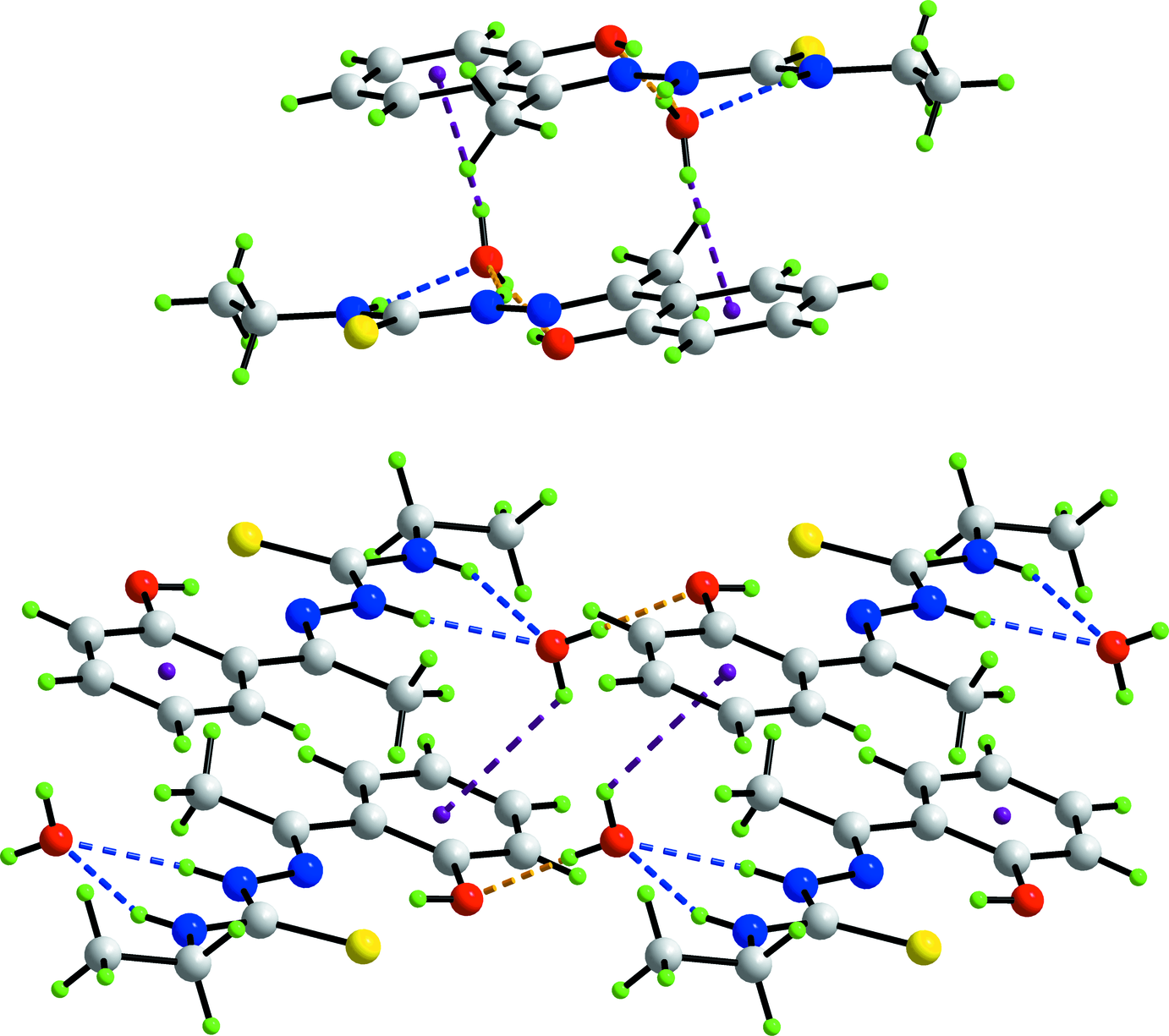

Supplement: Supplementary file 5 [file e-71-0o244-fig2.tif]

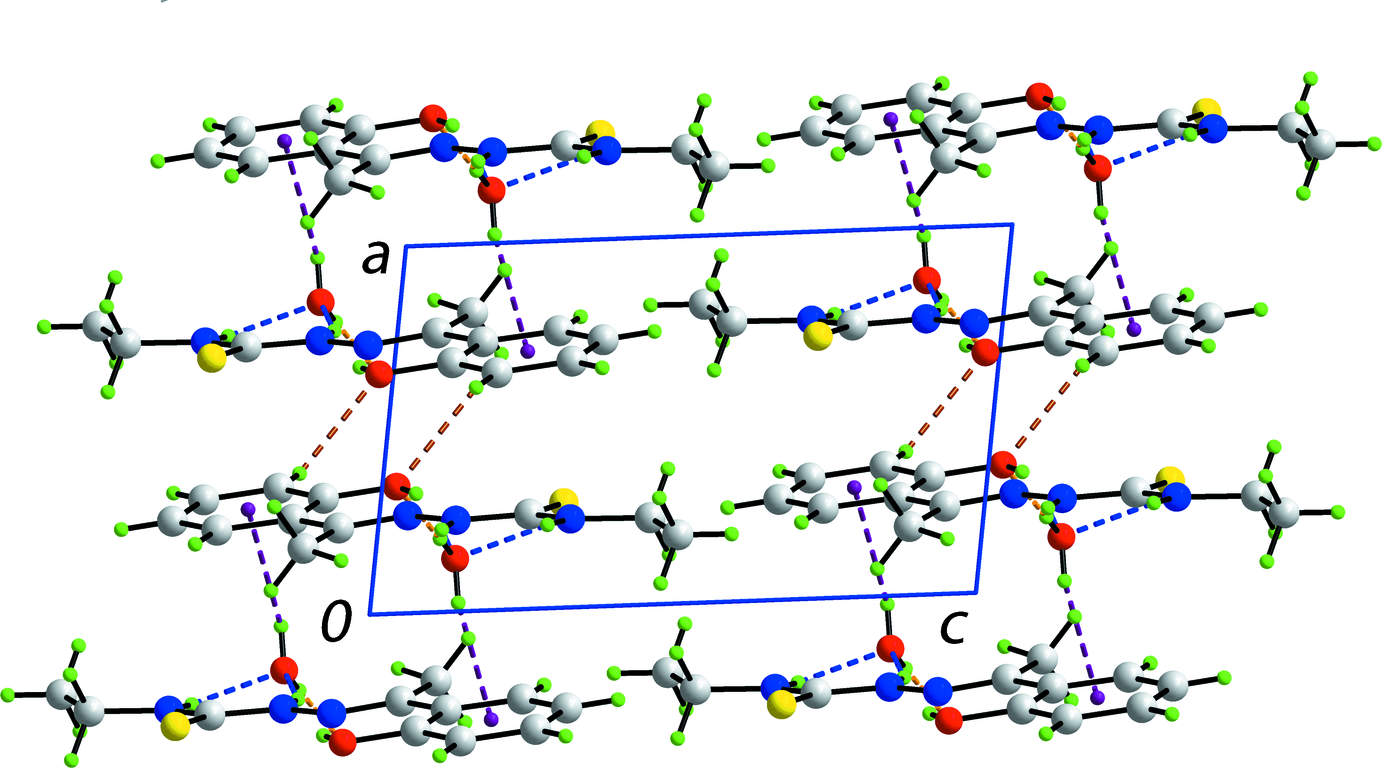

Supplement: Supplementary file 6 [file e-71-0o244-fig3.tif]
